# Supplementary material for: Accumulation of continuously time-varying sensory evidence constrains neural and behavioral responses in human collision threat detection
Source: PLoS Comput Biol. 2021 Jul 15;17(7):e1009096. doi: 10.1371/journal.pcbi.1009096 (PMC8282001; doi:10.1371/journal.pcbi.1009096)
Supplement: S1 Appendix — (PDF) [file pcbi.1009096.s001.pdf]

Supporting information appendix for

Accumulation of continuously time-varying sensory evidence constrains neural and behavioral responses in human collision threat detection

G. Markkula[1]*, Z. Uludağ[2,¤], R. M. Wilkie[2], J. Billington[2]

[1] Institute for Transport Studies, University of Leeds, Leeds, UK
[2] School of Psychology, University of Leeds, Leeds, UK
[*] Corresponding author at g.markkula@leeds.ac.uk
[¤] Current address: Department of Experimental Psychology, Ardahan University, Ardahan, Turkey

Stimulus perturbation

In the test track experiment reproduced here [1], small lateral oscillations and vertical vibration of especially the participant's own vehicle would have caused slight movement of the visual angle to the lead vehicle in the participant's field of view, making the looming detection task somewhat more challenging [2]. To mimic this effect in our paradigm, and also to further reduce any risk of pixel effects in our stimulus display, we introduced small random perturbations to the horizontal and vertical position of the stimulus image, computed as if arising from perturbations of the participant's viewpoint. These viewpoint perturbations were generated by passing white noise from a uniform distribution through digital Butterworth bandpass filters designed as specified in Table A, and scaled to the 95th percentile absolute amplitudes also listed in the table. The filter parameters for the horizontal and vertical perturbations were based on vehicle oscillation frequency spectra reported in [3] and [4], respectively. Initially we attempted using naturalistic values also for the amplitudes, but this resulted in subjectively excessive perturbations (possibly due to the lack of associated vestibular sensory perturbation), why the amplitudes were manually scaled until deemed subjectively appropriate by the researchers.

Table A. Stimulus perturbation parameters.

Direction	Lower stopband (Hz)	Passband (Hz)	Upper stopband (Hz)	Stopband attenuation (-)	Passband ripple (-)	95th %-ile absolute amplitude (m)
Horizontal	0 – 0.025	0.05 – 0.2	0.5 – 30	20	1	0.02
Vertical	0 – 0.25	0.5 – 4	5 – 30	10	1	0.01

Behavioral ANOVA

Table B provides the full results of the main behavioral ANOVA on the log-transformed optical expansion rate $\dot{\theta}$ at participant response. Besides the between-participant

differences, it is clear from the partial eta squared (η_p^2) that the two hypothesized main effects of initial car distance and acceleration magnitude explain most of the variance in the data. We will not discuss the full table of results here, but from a methodological point of view it is worth noting that there were small but statistically significant effects of experimental block and pre-looming wait time. The former was a minor (relative to the effects of initial distance and acceleration magnitude) increase in looming sensitivity over time, with mean $\dot{\theta}$ at response of 0.00213 rad/s and 0.00197 rad/s in the first and fifth blocks, respectively. The latter is discussed further below.

Table B. Results of repeated-measures ANOVA, with participant as a random factor, limited to first-order interactions only, and log($\dot{\theta}$) at response as the dependent variable.

Source	*SS*	*df*	*MS*	*F*	*p*	η_p^2
Experimental block	10.02	4	2.505	8.82	< 0.0001	0.04
Initial car distance	808.73	1	808.732	1255.48	< 0.0001	0.80
Acceleration magnitude	143.62	1	143.616	810.26	< 0.0001	0.42
Pre-looming wait time	5.00	4	1.249	19.75	< 0.0001	0.02
Participant	256.62	21	12.220	11.99	< 0.0001	0.57
Experimental block × Initial car distance	0.32	4	0.079	1.49	0.203	< 0.01
Experimental block × Acceleration magnitude	0.98	4	0.245	4.60	0.001	< 0.01
Experimental block × Pre-looming wait time	1.15	16	0.072	1.35	0.155	< 0.01
Experimental block × Participant	24.19	84	0.288	5.40	< 0.0001	0.11
Initial car distance × Acceleration magnitude	1.19	1	1.189	22.30	< 0.0001	< 0.01
Initial car distance × Pre-looming wait time	0.40	4	0.099	1.87	0.114	< 0.01
Initial car distance × Participant	13.69	21	0.652	12.24	< 0.0001	0.06
Acceleration magnitude × Pre-looming wait time	0.03	4	0.009	0.160	0.957	< 0.01
Acceleration magnitude × Participant	3.76	21	0.179	3.36	< 0.0001	0.02
Pre-looming wait time × Participant	5.32	84	0.063	1.19	0.117	0.03
Error	197.08	3698	0.053			
Total	1510.83	3972				

Approximate priors from the test track study results

To obtain reasonable initial model parameter ranges for both the ABC and MLE model fits, we performed a first ABC fit of each model to the information available from the Lamble et al. test track study [1]; specifically their observed mean $\dot{\theta}$ at detection for the two looming conditions with 0.7 m/s^2 lead vehicle deceleration, and the width of the corresponding confidence intervals (the latter approximately estimated from their Figure

4). We used the same basic rejection sampling algorithm as described for the main model fits in Materials and Methods, running 300,000 simulated replications of the test track experiment per model, drawing from wide uniform priors. We rejected ABC samples for which any of the four normalized deviations $(x_{\mathrm{sim}} - x_{\mathrm{obs}})/x_{\mathrm{obs}}$ between simulation and observation of the mean $\dot{\theta}$ or its confidence interval width was greater than 0.5, or for which there was more than 5 % premature looming detection responses in either of the looming conditions (Lamble et al. do not mention observing any such early responses). The equal-tailed 95 % credible intervals of the posterior distributions obtained in this way were expanded in both directions by 50 % of their width (but limited to positive values), to obtain the parameter ranges that we then used in the main model fits, to bound the uniform ABC priors and the MLE grid search. The reasons we adopted these relatively permissive ABC distance metric thresholds and the subsequent widening of the credible intervals were that (i) we were not certain how similar behavior would be between the test track and laboratory paradigms, and (ii) the test track means were averages across participants, whereas we wanted to fit our models to individual participants, so we needed to allow for between-participant variability. The final values of the hyperparameters mentioned above were arrived at by some trial and error to avoid obvious truncation of the main ABC or MLE model fits; the conclusions reported in this paper were robust to these hyperparameter variations. Examples of the adopted priors can be seen in Fig II, as the ranges within which the posterior distributions are plotted.

Per-participant model fits

Fig A shows per-participant MLE fits for the T, A, and AV models, for five example participants. Participant "mllh" (rightmost column) is included as the only participant for which the threshold model came close to the performance of the accumulator model (cf. Fig 2B in the main text); the other four participants were chosen at random.

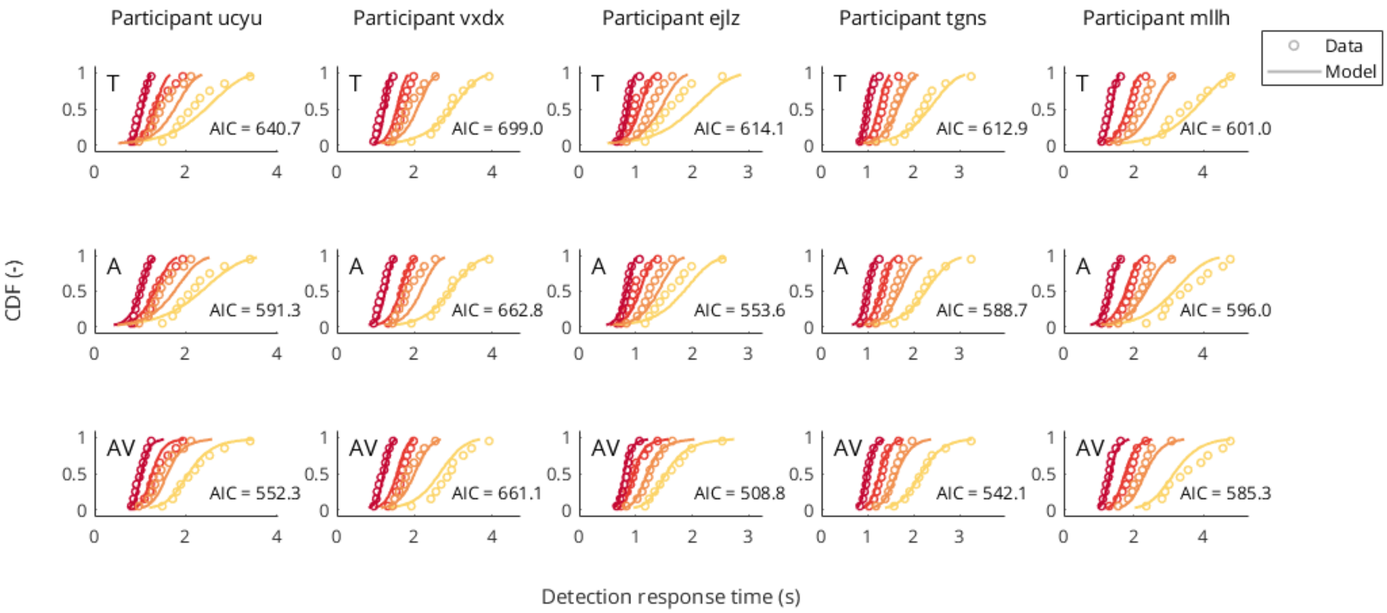

Fig A. Example per-participant fits. As Fig 2A in the main text, but for individual participants. Lower AIC values indicate a preferable model.

Model parameter estimates

Fig B shows the parameter values estimates obtained from the ABC and MLE fits of model AV. The Bayesian posteriors are combined across all participants. It can be noted that the two fitting methods yielded comparable parameter estimates, i.e., the per-participant MLE values fall predominantly within regions that are of high posterior probability according to the ABC fits. The cross-participant average MLE-fitted parameter values were $T_{\mathrm{ND}} = 0.324$ s, $\sigma = 0.202$, $K = 1390$, $\sigma_K = 0.302$.

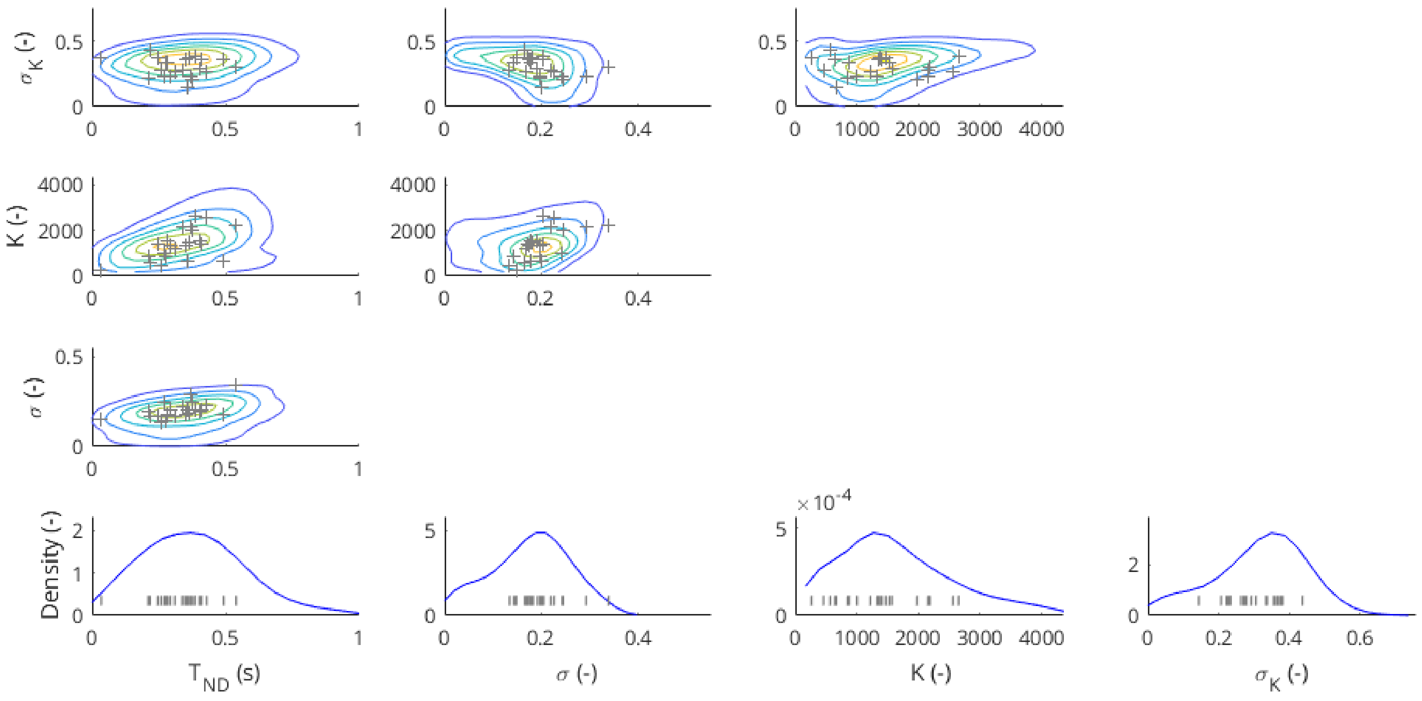

Fig B: Obtained parameter values for the variable-gain accumulator model (AV). The bottom row of panels shows marginal cross-participant Bayesian posteriors (curves) and per-participant maximum likelihood estimates (MLEs; markers along bottom) for each model parameter, and the upper rows of panels show the same for all combinations of two model parameters (contours and markers, respectively). Slight jitter has been added to the gridded MLE parameter values to make them distinguishable from each other. The parameter ranges shown here correspond to the ranges of the uniform priors obtained as described above in this document.

Effect of pre-looming wait time on collision threat detection

As mentioned above, the behavioral ANOVA identified a statistically significant effect of pre-looming wait time (the time delay of 1.5-3.5 s between appearance of the lead vehicle and its deceleration onset) on the optical expansion rate at detection response. The left panel of Fig C visualizes this effect, showing that for longer pre-looming wait times, participants tended to respond at lower optical expansion rates, i.e., sooner after looming onset.

In our model fits (both ABC and MLE) the variation in pre-looming wait times were included in the simulated experiments that were generated for each tested model

parameterization. However, as described in Materials and Methods, the goodness of fit of the models was tested by comparing observed and model distributions per looming condition, thus pooling across the pre-looming wait times. In other words, the models were not fitted to reproduce the pre-looming wait time effect. However, as can be seen in the right panel of Fig C, model AV did nevertheless reproduce this effect rather closely, including the hint of an interaction with looming condition (a non-significant trend in our behavioral analyses; Table II). The reason model AV exhibits this behavior is that before looming onset (while $\dot{\theta} = 0$) it will accumulate some noise, and with longer pre-looming wait times it is more likely to randomly be close to the decision threshold once looming begins increasing.

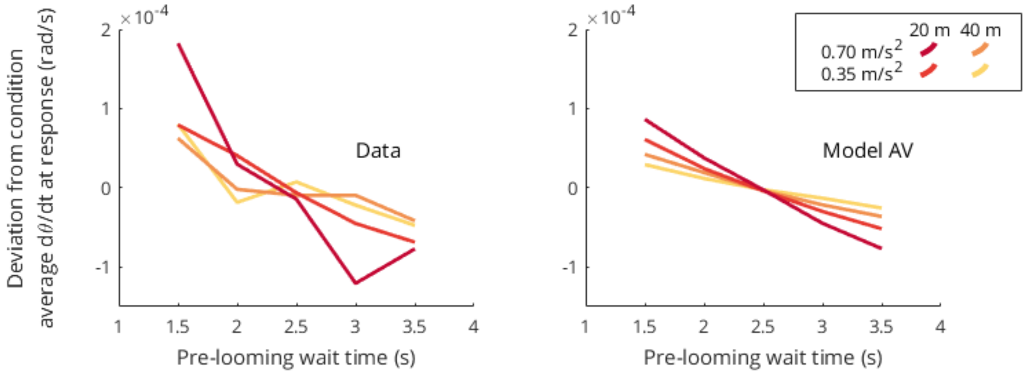

Fig C. Effect of pre-looming wait time on collision threat detection. Observed and model-predicted (model AV, MLE-fitted) average optical expansion rate at detection response, across the five different pre-looming wait times, separately for each looming condition. For ease of comparison across looming conditions, the per-condition averages have been deducted, such that the plots effectively show average deviations from per-condition averages.

Alternative model variants

In addition to the looming accumulator model variants mentioned in the main text, defined as in Equation (1), we also tested a number of other accumulator models, based on the following more general formulation:

$$E(i) = \max\left(0, E(i-1) + \left[-\frac{1}{\tau}E(i) + \tilde{K}\left(\dot{\theta}(i) - \dot{\theta}_s\right)\right]\Delta t + \sigma\nu(i)\sqrt{\Delta t}\right), \quad \text{(S1)}$$

where τ is a leakage (memory decay) time parameter and $\dot{\theta}_s$ is a gating threshold on the sensory input, essentially preventing accumulation of looming evidence until $\dot{\theta}(i) > \dot{\theta}_s$, since we constrain $E(i) \geq 0$. Note that with τ large and $\dot{\theta}_s = 0$, Equation (S1) reduces to Equation (1).

Using MLE, we tested models AG and AL with just the gating and leakage assumptions added beyond the basic accumulator model, but as shown in Fig D neither of them provided better fits of the data than the AV model (ΔAIC positive for all participants, as seen in rightmost panel).

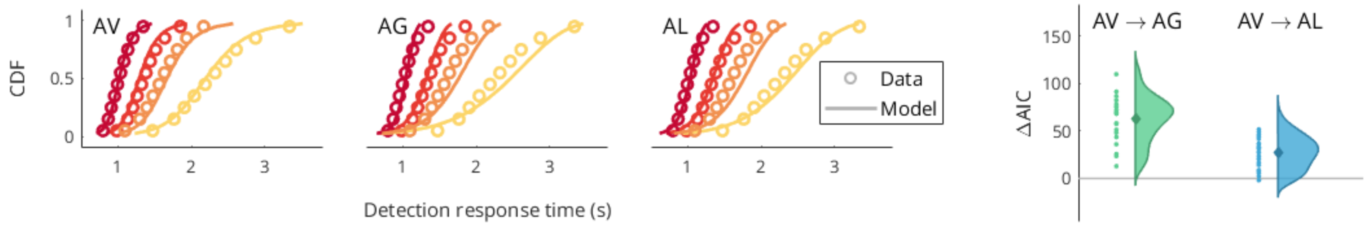

Fig D. Maximum likelihood estimation fits of alternative accumulator models. See Fig 2 in the main text for explanations. Here, the models shown are the accumulator models extended with either variable gain (AV; repeated from Fig 2), sensory input gating (AG), or evidence leakage (AL). Positive ΔAIC values indicate preference for the former model in the comparison.

We further investigated these alternative accumulator models using ABC, with which it was computationally feasible to also test even more complex model variants, obtained by further combining the assumptions of gain variability, gating, and leakage. Fig E shows, for each tested model m, the geometric mean $gBF_{\mathrm{A},m}$ of the per-participant Bayes Factor $BF_{\mathrm{A},m} = \frac{p(\mathrm{A}|\mathrm{data})}{p(m|\mathrm{data})}$ for the comparison between models A and m across different values of the ABC distance threshold ϵ_{RT}. Fig E shows only model comparisons for which at least 15 participants had at least 100 non-rejected ABC samples; for $\epsilon_{\mathrm{RT}} < 0.3$ s this was true for no model comparison. It can be noted that even though the obtained Bayes factors varied with ϵ_{RT}, the ordering of the models was robust to the choice of ϵ_{RT}. The ABC fits align with the MLE fits in ordering AV above AL, and AL above AG. Overall, both the MLE and ABC results provide rather compelling evidence against the inclusion of gating in the accumulator model; this can be taken as a further argument against the assumption that fixed sensory thresholds play a role in collision threat detection. The results are somewhat less clear with respect to the leakage assumption. The MLE results in Fig D do suggest that model AV is preferable to model AL, but given what was already mentioned in the main text about our broad ABC priors potentially over-penalizing model complexity, we would not strongly argue that model AV is also preferable to model AVL based on the results shown in Fig E. Conversely, however, our model comparison clearly does not provide evidence for favoring AVL over AV. In fact, also our finding that looming sensitivity increased with pre-looming wait time (Fig C) could be taken as indirect evidence against leakage playing a strong role in this paradigm, since leakage counteracts the type of pre-looming accumulation of noise that was discussed above.

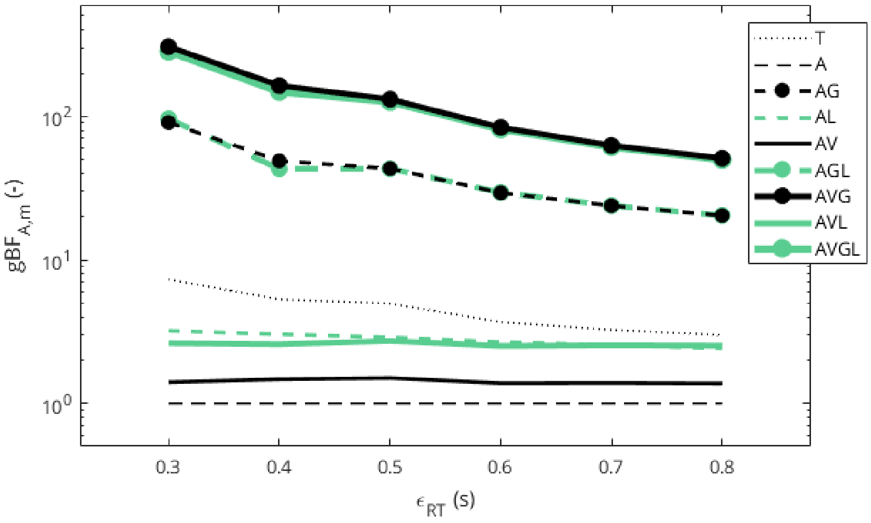

Fig E. Model comparison with Approximate Bayesian Computation (ABC). Geometric mean $gBF_{\mathrm{A},m}$ of per-participant Bayes Factors, for comparisons between the basic looming accumulator model A and the other tested models, as a function of the ABC distance threshold ϵ_{RT}. A value of $gBF_{\mathrm{A},m} > 1$ indicates evidence in favor of model A over the alternative model.

Impact of EEG preprocessing choices on ERPs and CPP onset estimates

To eliminate concerns that the obtained CPP signature might have arisen from excessive filtering of low EEG frequencies or excessive exclusion of ICA components as ocular artefacts, the ERP analyses were rerun without these processing steps. Fig F illustrates the impact of just the 0.1 Hz high-pass filter (compare panels (i) and (iii) of Fig F) and of the ICA-based ocular artefact removal (compare Fig F(i) vs Fig 3C in the main text). The ocular artefact removal reduces the overall amplitude of the ERP somewhat, seemingly due to the presence of time-varying within-trial eye artefacts, i.e., on the same time scale as the CPP. The high-pass filter introduces a slight suppression into negative voltages before the CPP build-up commences. However, it is clear that neither of these EEG preprocessing steps change the qualitative aspect of the response-locked CPP signature; it remains late, rapid, and without a clear effect of looming condition on CPP duration.

Panels (ii) and (iv) of Fig F show the results of applying the same CPP onset estimation algorithm as described in the main text to these differently pre-processed ERP data. Removing the high-pass filter (panel (iv)) introduces a small but noticeable separation in the CPP onsets between looming conditions (74 ms maximum difference between condition means, with upper edge of 95 % confidence interval at 136 ms). On closer inspection, however, all or most of this effect arguably seems to be artefactual in nature, deriving from our CPP onset estimation method, which defines CPP onset as occurring when the ERP last exceeds 30 % of the ERP amplitude at response, an amplitude which in turn is dependent on looming condition, as described in the main text, thus effectively setting the onset threshold at different voltages for the different conditions. We adopted this method because it is simple, allows estimation of CPP onset regardless of the at-response ERP in the specific trial, and since with the high-pass filter's suppression of the ERP into negative voltages before CPP build-up, 30 % of the positive ERP peak occurs at

a point in time where the ERP is changing rapidly, such that there is little impact on the onset estimation of the between-condition difference in CPP onset threshold. Without the high-pass filter, this between-condition difference in threshold makes a more noticeable difference. Panel (v) in Fig F shows the results of alternatively defining CPP onset as occurring at 30 % of the average ERP at response across all trials for the participant in question, i.e., with the same threshold for all trials. This method does indeed yield smaller differences between looming conditions (35 ms maximum difference between means, with upper edge of 95 % confidence interval at 106 ms), but as expected the estimated distributions are no longer well-formed, due to ERP being below threshold at the response itself on many trials.

Overall, it may be argued that while the high-pass filter distorts the visual aspect of the CPP signal somewhat, this filtering is useful for estimating the CPP onsets. Future work should improve on these methods, to allow non-biased CPP onset estimation also without prior high-pass filtering. Crucially, however, it should be noted that even if the results shown in panel (iv) of Fig F are taken at face value, they can still not be seen as suggesting that the CPP signal in our paradigm aligns with the evidence signal in our behavioral model, since the 74 ms difference between conditions in panel (iv) is still an order of magnitude smaller than the 1.3 s maximum between-condition difference in response times. (Also, as shown in Fig I(ii) further below, using these CPP onsets for model fitting does not change the conclusions from the comparison between models predicting CPP onset.)

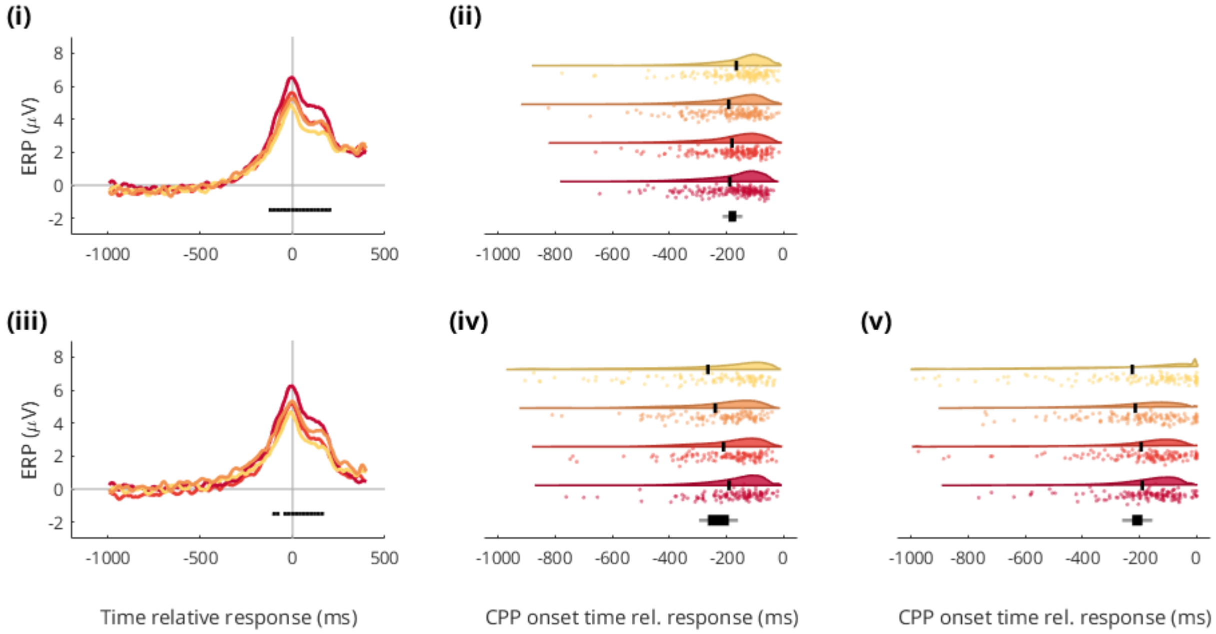

Fig F: Impact of EEG preprocessing choices on CPP signature and estimated onsets. (**i**) The same response-locked ERPs as in Fig 3C of the main text, but without ICA ocular artefact removal. (**ii**) CPP onsets, as in Fig 3F of the main text, but obtained from the ERP data shown in panel A of this figure. (**iii**) Exactly the same as Fig 3D in the main text, i.e., response-locked ERPs without both 0.1 Hz high-pass filtering and ICA ocular artefact removal. (**iv**) CPP onsets obtained using the same method as previously, from the ERP data shown in panel C of this figure. (**v**) CPP onsets for the data in panel C, when adjusting the estimation method to use an absolute ERP threshold rather than a threshold relative to ERP at response.

Estimation and modeling of CPP onsets

Fig G shows response locked ERPs for all 22 participants analyzed in this study, highlighting the five participants for which our method for CPP onset estimation did not produce useful results (method and resulting trial exclusions described in Materials and Methods). For participant emlq this was due to the peak ERPs at response occurring near or below baseline levels, for the other four of these participants there was no noticeable CPP peak (low effect size in ERP amplitude change during the last 500 ms before response).

Fig H shows the result of a further sensitivity analysis of the CPP onset estimation method, testing all combinations of (i) the number of sorted trials averaged across, varied in {3, 5, 7}, (ii) the minimum at-response ERP required for inclusion of such an averaged trial, varied in {0.15, 0.2, 0.25} μV, and (iii) the fraction of at-response ERP at which the CPP was estimated as having its onset, varied in {25, 30, 35} %. The figure shows that the CPP onset estimation method was robust to these parameter variations, in the sense that the same five excluded participants mentioned above consistently caused around half of the exclusions of averaged ERP trials (left panel), and the estimated CPP onset occurred close in time to the overt response (middle panel), with small differences between looming conditions (right panel).

Fig I(i) complements Figs 3G and H in the main text, by illustrating the results of MLE-fitting also models T and A to the CPP onset distributions. Fig I(ii) shows the results of MLE-fitting the same models to the CPP onset distributions in Fig F(iv), to show that even if taking these—as explained above arguably biased—CPP onset estimates at face value, the conclusions from the model comparison remain unchanged.

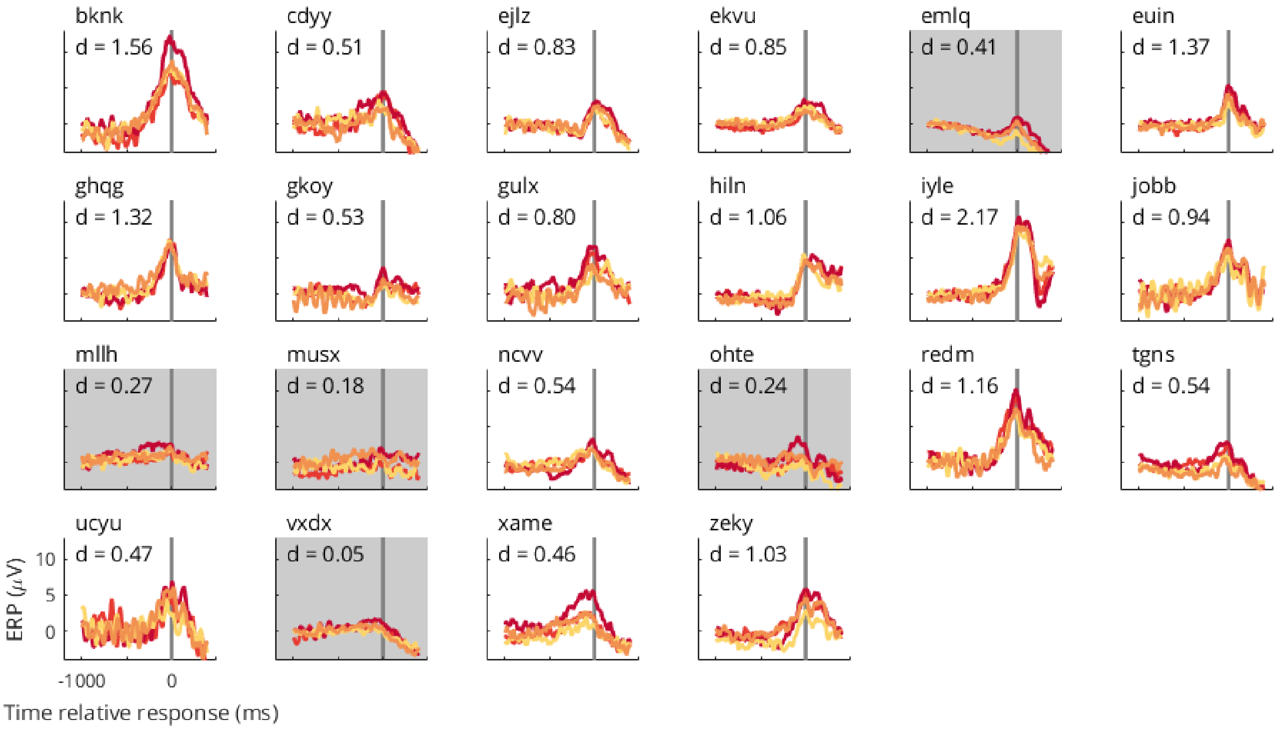

Fig G: Response-locked ERP data per participant. As the top panel of Fig 3 in the main text, but for each participant individually (identified by four-letter strings). Also shown is the Cohen's d effect size for the comparison between per-participant grand average 500 ms before response and at response. The five highlighted participants did not permit reliable CPP onset estimation.

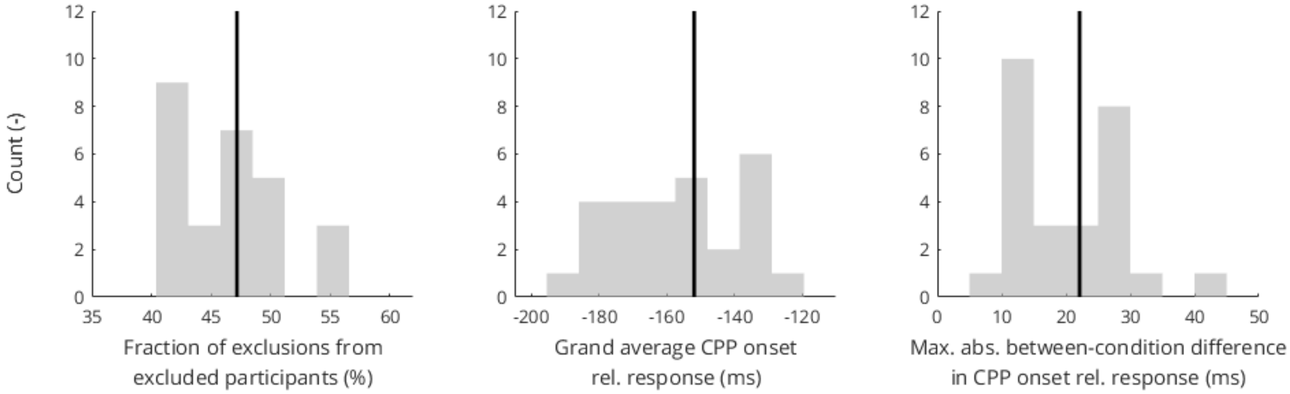

Fig H: Sensitivity analysis of CPP onset estimation method. For each metric, the black line shows the value reported in the main text, and the histogram shows the variation around this value obtained when varying the parameters of the CPP onset estimation method.

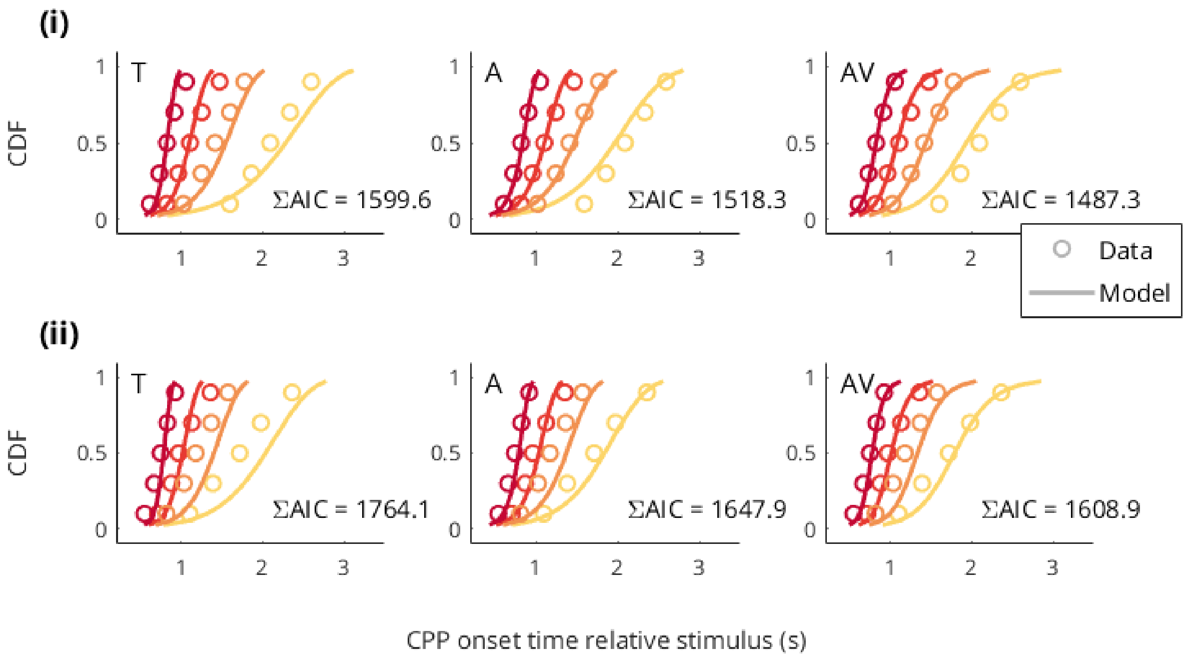

Fig I: MLE model fits to CPP onsets. (**i**) As Fig 3G in the main text, but also showing results for models T and A. ΣAIC is the total AIC for the model, across all participants, with a lower value indicating a preferable model (see Fig. 3H for confidence intervals for the differences in ΣAIC). (**ii**) Results when instead fitting these models to the CPP onset estimates shown in Fig F(iv).

We wanted to ascertain whether there existed parameterizations of the accumulator models which could achieve good fits of our behavioral data, while at the same time also exhibiting evidence build-up profiles more similar to our CPP observations, where onset and between-condition separation occurred late in the trial. We therefore extended our per-participant ABC fits with an additional distance metric: For each observed or model-simulated trial i we took the response-locked build-up profile $e_i(t)$, either the response locked ERP or model evidence $E(t)$. In the latter case, we needed to determine how much of the non-decision time T_{ND} to allocate to early sensory and late neuromuscular delays, respectively; we therefore introduced an additional model parameter α_{ND} representing the fraction of T_{ND} occurring before the evidence accumulation, with a uniform prior in [0, 1]. We then calculated the grand mean $\bar{e}_{\mathrm{R}}$ of $e_i(t)$ across all trials, in the last 100 ms before the overt response, to quantify the grand-average at-response peak amplitude. For each condition j we calculated the mean of $e_i(t)$ for trials in condition j, in the interval 400-700 ms before response, and calculated the fraction $f_j = \bar{e}_{j,0}/\bar{e}_R$, to quantify the amount of early build-up in condition j. We then extended our ABC rejection sampler to retain model parameterizations for which not only the previously described ϵ_{RT} threshold criterion was achieved, but also where the difference between observed and model-simulated f_j was smaller than a threshold ϵ_f for all looming conditions. Fig J shows the results of purely behavioral fits ($\epsilon_f = \infty$) of model AVL for ϵ_{RT} at 0.4 and 0.8, as well as fits where we progressively reduced ϵ_f from 0.8 in steps of 0.1, to find the smallest ϵ_f for which at least 100 ABC samples were retained. Included here are only the 17 participants who showed clear CPP profiles as described above, minus a further exclusion of two participants for which 100 ABC samples were not retained with ($\epsilon_{\mathrm{RT}} = 0.4$, $\epsilon_f = \infty$); more permissive inclusion criteria yielded similar results. It is clear from the figure that

the looming accumulator model is not able to achieve the type of late build-up profile observed in our CPP data, not even when setting a permissive behavioral threshold $\epsilon_{\mathrm{RT}} = 0.8$, at which the response time fits become noticeably corrupted. We are showing the AVL model here since it is the most complex of the models with good behavioral fits, and thus the model with best chances of being flexible enough to also account for the neural data. The corresponding figure for model AV is very similar to Fig J.

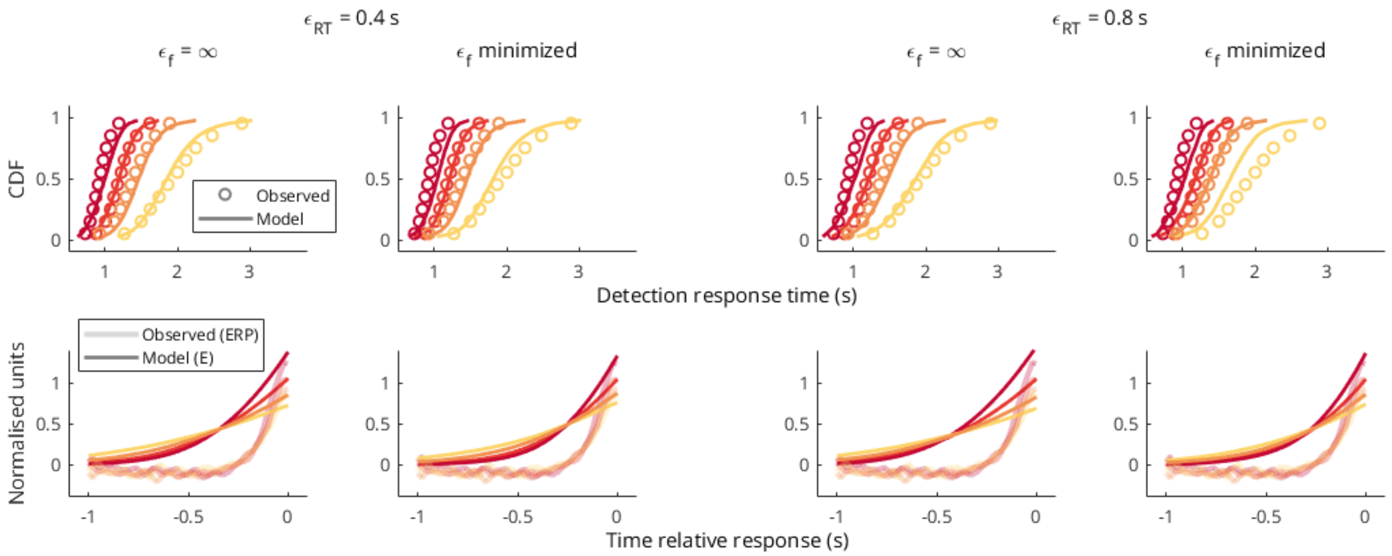

Fig J: Testing the accumulator model's ability to also account for the neural observations. "Vincentized" cumulative distributions of detection response time (top panels) and normalized grand averages of observed response-locked ERP and model evidence $E(t)$, for ABC fits of model AVL. Looming conditions are color coded as in the figures in the main text. The leftmost and rightmost two columns show fits obtained with the ABC distance threshold for response times set to $\epsilon_{\mathrm{RT}} = 0.4$ s and $\epsilon_{\mathrm{RT}} = 0.8$ s, respectively. In the fits shown in the second and fourth columns, also a threshold ϵ_f was imposed, for a metric quantifying the distance between the model evidence trace and the observed ERPs.

Panel (i) in Fig K illustrates a hypothetical two-stage accumulation process which could possibly explain both our behavioral and neural observations. The looming evidence integrated in the looming accumulator model $\dot{\theta}(t) \mapsto E(t)$, corresponding to the computational models proposed in this paper, is passed, after thresholding, to a second accumulator which decides on the overt response. Panel (ii) in Fig K shows, as cross-participant averages per looming condition, the result of taking the evidence $E(t)$ in the MLE-fitted model AV, passing it through a logistic sigmoid threshold centered at $E(t) = 1$, i.e., $1/(1 + e^{-k(E(t)-1)})$, with $k = 20$, and then subjecting the output of this thresholding to a second integration to yield a second evidence quantity $E'(t)$. As can be seen, $E'(t)$ shows similar condition-independent late onset and condition-dependent separation in at-response amplitude (and hence also in build-up rate) as we observed for the CPP in our paradigm. This type of model can of course be tested formally, but is substantially more complex (at least k and a separate decision threshold are needed as free parameters, and possibly also a separate noise intensity); therefore such an investigation is left outside of the scope here.

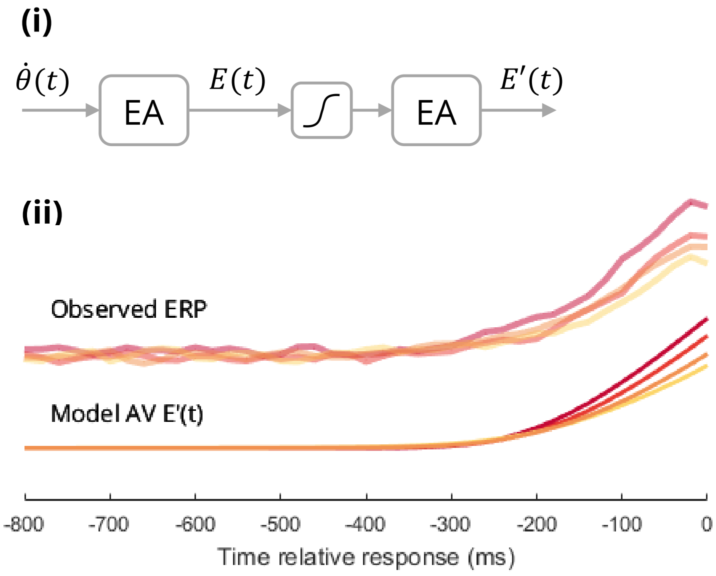

Fig K. Two-stage evidence accumulation. (**i**) Schematic illustration of the concept, with two evidence accumulators (EAs) in sequence, and a sigmoid threshold applied to the output of the first EA to generate the input for the second. (**ii**) Observed response-locked ERPs (same as in the top panel of Fig 3C in the main text) and the second-stage accumulator evidence $E'(t)$, when using the same first-stage accumulator evidence $E(t)$ as shown for model AV in Fig 3E in the main text.

As mentioned in the main text, separations in CPP peak amplitude at overt response has been previously reported, between experimental conditions of different saliency [5]–[7]. This separation has been suggested to arise either due to interference from motor preparation-related negative potentials at frontocentral electrodes [6] or due to continued evidence accumulation after the decision threshold has been reached, before feedback on the motor execution has been fed back to the CPP source [7]; the model evidence traces in the lower panel of Fig 3E in the main text provide an illustration of this latter idea. This post-decision separation in $E(t)$ between conditions in the looming accumulator, fed through the sigmoidal threshold, is what causes the separation in $E'(t)$ seen in panel (ii) of Fig K. However, the two-stage model as such does not fundamentally imply at-response separation in accumulated evidence; e.g., for lower values of k the model predicts small or no such separation.

References

[1] D. Lamble, M. Laakso, and H. Summala, ‘Detection thresholds in car following situations and peripheral vision: implications for positioning of visually demanding in-car displays’, *Ergonomics*, vol. 42, no. 6, pp. 807–815, Jun. 1999, doi: 10.1080/001401399185306.

[2] M. Gould, D. R. Poulter, S. Helman, and J. P. Wann, ‘Detection of vehicle approach in the presence of additional motion and simulated observer motion at road junctions.’, *Journal of Experimental Psychology: Applied*, vol. 19, no. 2, pp. 171–184, 2013, doi: 10.1037/a0033286.

[3] J. Östlund *et al.*, ‘Driving performance assessment - methods and metrics’, AIDE Project Deliverable D2.2.5, 2005. Accessed: Aug. 31, 2020. [Online]. Available: http://www.aide-eu.org/res_sp2.html.

[4] Y. Qiu and M. J. Griffin, ‘Transmission of vibration to the backrest of a car seat evaluated with multi-input models’, *Journal of Sound and Vibration*, vol. 274, no. 1–2, pp. 297–321, Jul. 2004, doi: 10.1016/j.jsv.2003.05.015.

[5] R. G. O’Connell, P. M. Dockree, and S. P. Kelly, ‘A supramodal accumulation-to-bound signal that determines perceptual decisions in humans’, *Nat Neurosci*, vol. 15, no. 12, pp. 1729–1735, Dec. 2012, doi: 10.1038/nn.3248.

[6] S. P. Kelly and R. G. O’Connell, ‘Internal and External Influences on the Rate of Sensory Evidence Accumulation in the Human Brain’, *Journal of Neuroscience*, vol. 33, no. 50, pp. 19434–19441, Dec. 2013, doi: 10.1523/JNEUROSCI.3355-13.2013.

[7] Y. Boubenec, J. Lawlor, U. Górska, S. Shamma, and B. Englitz, ‘Detecting changes in dynamic and complex acoustic environments’, *eLife*, vol. 6, p. e24910, Mar. 2017, doi: 10.7554/eLife.24910.
