# Supplementary material for: Accumulation of continuously time-varying sensory evidence constrains neural and behavioral responses in human collision threat detection
Source: PLoS Comput Biol. 2021 Jul 15;17(7):e1009096. doi: 10.1371/journal.pcbi.1009096 (PMC8282001; doi:10.1371/journal.pcbi.1009096)
Supplement: Attachment — Submitted filename: Response to reviewers - v1.1.pdf [file pcbi.1009096.s002.pdf]

Below the editor and reviewer comments are reproduced, together with responses from us in blue.

In our revised submission, we are including one clean version of the revised manuscript, and one version where the changes since the initial submission are highlighted.

Editor comments:

All reviewers indicate they find this an interesting paper, with an interesting application of accumulator models to a real-life situation. They also all have some concerns. I agree with these concerns, which in my main boil down to the following issues that need to be addressed:

We provide brief notes here; see our responses to the reviewer comments for the details.

1) to what extent is this really a change detection problem rather than a two-alternative forced choice decision, and how does this impact the modeling? I would like to see, if possible, the suggested single-threshold model Reviewer 1 is suggesting

We agree with you and Reviewer 1 that this is a change detection problem rather than a two-alternative forced choice decision; our model actually is a single-boundary model already precisely for this reason. As we explain below, we are not the first to apply this general type of model to tasks of a change detection nature. With respect to the further simplified type of single-boundary model mentioned by Reviewer 1, if we understand the suggestion correctly we do not think it can be meaningfully applied to our task, where the sensory evidence varies continuously over time.

2) exactly how do the situational variables map onto the model parameters, and could other mappings also be possible, as Reviewer 2 is suggesting?

We have added further clarifications to the manuscript about the situational parameters, as well as about model flexibility and generalization (we interpret your comment as relating to this comment from Reviewer 2).

3) is it possible to estimate the CPP onset with certainty, as Reviewer 1 asks? And could the CPP become more "normal" when a different high-pass filter is used, as Reviewer 3 suggests?

We agree that it is not obvious how to define and estimate CPP onset. We have now carried out additional sensitivity analyses of our CPP onset estimation method, showing that our findings of a late and condition-independent (relative to response) CPP in our paradigm is robust to variations in the parameters of our method. Also the point about the high-pass filter was very valid; we now show that the nature of our CPP signatures is not a result of this filtering.

4) a final, more theoretical problem, related to issue 1), is how does the accumulator model "know" when to start?

This is indeed a challenging problem, and to understand it better we have dug deeper into our behavioral results and model fits. Our conclusion is that it seems the participants, like our models, began accumulating evidence already from the start of each trial (when the lead vehicle stimulus first appears), 1.5-3.5 seconds before the onset of visual looming. We found that our accumulator model without gating or leakage correctly predicts an effect of the duration of this waiting time. This was not an effect that the model was fitted to reproduce, so our results here can be seen as providing additional support for our model.

Reviewer #1 comments

The authors examine a model of collision threat detection by humans. They contrast an evidence accumulation model of this capability against a simple threshold model without evidence accumulation. The evidence accumulation style of model wins hands down. That model comparison seems quite compelling. They also examine the centroparietal positivity (CPP) computed from EEG recordings of the participants during task performance. In their work, the CPP does not appear to reflect an evidence accumulation process, but rather something closer to decision readout perhaps. This is in contrast to the results of Kelly & O'Connell and colleagues, who established a relationship between evidence accumulation and the CPP. The stimuli here (looming vehicles), however, might not be processed in quite the same way as less naturalistic stimuli. Still, there was a CPP, and the absence of correlation with evidence accumulation is noteworthy.

I found the paper to be interesting, novel, and of obvious practical importance.

Many thanks for your review, and for your kind words here.

I have a few larger and more minor points of concern.

Major issues:

1) The experimental situation isn’t just different from standard perceptual tasks like dot motion discrimination in terms of the nature of the stimulus, but also in terms of the types of choices. This task does not involve two choices. It involves only one, and the question is when to choose it. This is more like a change-detection task than the two-choice type of task usually addressed by the diffusion model, isn't it? How does a change-detection model compare to the diffusion model? I believe change-detection algorithms such as the CUMSUM test are accumulators too, but without a lower threshold. This is not necessarily a bad thing, but it just makes the mapping to the decision literature a little less straightforward. Isn't this really a change-detection problem? I think the authors ought to consider that.

You are correct that change-detection is definitely not the most common type of paradigm for evidence accumulation / drift diffusion modeling. There are a couple of precedents before us, however. Thanks for pointing us to CUSUM – we were not familiar with it, and we agree with you that our model—and the previous ones of intermittently changing abstract stimuli—effectively implements the CUSUM technique. In response to your comment on these matters, we have added the following sentence to the section "A visual looming…" in Results:

"It may be noted that our model, like previous evidence accumulation models of detection of intermittent, subtle changes in abstract stimuli [14,16], effectively implements Page's cumulative sum ('CUSUM') technique for change detection [61]."

2) Further, there are applications of single-boundary diffusion models to, among other things, interval timing (Simen et al., 2016). The nice thing about a single boundary model is that it has a very simple, closed-form expression for the first-passage time (or decision time) distribution, which is the inverse Gaussian distribution. This is much easier to compute than the first-passage time distribution of a two-boundary model. Much faster than approximate Bayesian computation too. It might be worth fitting to the data.

Our drift diffusion model actually has just a single boundary. To emphasize this further, we have now added the keyword "single-boundary" where the model is first introduced, in the section "A visual looming..." in Results.

However, despite the model being single-boundary, as far as we are aware there is no closed-form expression for the distribution of its first-passage times, since the drift rate is continuously time-varying. To clarify this point, we have added the following sentence to the same section as just mentioned:

"Due to the time-varying drift rate in our accumulator model, there is no closed-form expression for its response time distribution [62]; we estimated these distributions numerically instead."

3) It's not clear to me how the authors embedded the assumption of a declining threshold within trials into their model fits. What was the shape and equation for that function? On p. 13, the text suggests there was not in fact a declining threshold. It was fixed at 1. So how does that square with the description in Fig. 1? The text should describe how the declining threshold was modeled, or why it wasn't, given Fig. 1.

We did not include a declining threshold in our model. We are not sure what part of the Fig 1 caption gave that impression, but to hopefully remedy this we have added the keyword "fixed" to the following sentence in that caption:

“This model posits that the visual looming evidence shown in panel A is integrated over time together with normally distributed noise, up to a fixed threshold at which detection occurs”

4) Need a table of the model parameters and their fitted values and any other info needed (hyperparameters?). I think one should always display that in publications that use this type of model.

We agree that it is important to share fitted parameter values. The full overview of model parameters across participants is provided by Fig S2; we do not see an obvious way to summarize this information in a table. Instead, to address your comment we have added mention of numerical values for cross-participant averages of the maximum-likelihood fits next to Fig S2, and to be clearer about the free parameters in the main text we have added the following sentence to the section “A visual looming accumulator…” in Results:

“Model AV had four free parameters: the non-decision time T_{ND}, the accumulator noise intensity σ, and mean and standard deviation K and σ_K of the looming input gain; estimated values for these parameters across participants are shown in Fig S2.”

With respect to hyperparameters, we have gone through the Materials and Methods again to verify that these are all appropriately listed there.

5) I'm concerned about excessive reliance on the *onset* of CPP activity as the main dependent variable in the EEG section of the paper. Maybe that’s not the best feature of the CPP to examine, particularly when it would seem that onset-estimates might be sensitive to the way in which they are computed (?).

We are not sure we would agree that we rely excessively on CPP onset estimates; we also analyse ERP amplitudes both in terms of maximum amplitudes and the times at which the amplitudes differ between conditions (Fig 3; section “Looming accumulation explains…” in Results). However, we do agree with you that our results could be sensitive to the exact method for estimating CPP onsets. To alleviate this concern (and also to address related comments from Reviewer #2’s), we have now carried out a sensitivity analysis of our method, reported on in the Supporting Information, and mentioned in the Data acquisition and preprocessing section of Materials and Methods:

“Since our CPP onset estimation method was novel, and not originally planned for, we conducted a sensitivity analysis. As illustrated in Fig S8, this analysis showed that the obtained CPP onset estimates were robust to variations in the parameters of our method.

As you will see in the Supporting Information, this analysis shows that despite variations in the parameters of our method, the estimated average CPP onset was

always close in time to the overt response, with minimal variation between looming conditions (if any), and the same participants always contributed the lion's share of the data that we had to exclude. In other words, the main takeaways from the CPP onset estimation did not change.

It seems from the figure that buildup rate and maximum amplitude clearly distinguish the conditions.

Yes, you are correct that in the late stage (final ~300-400 ms before response) in which CPP build-up is happening in our paradigm, there is indeed separation between the conditions. This statistically significant effect of looming condition is indicated by the dots along the bottom of the top panel in Fig 3C, and the analysis in question is described in Statistical analysis in Materials and Methods. See also the in-depth presentation of the 'two-accumulator' model in the Supporting Information (Fig S11), where one of the takeaway messages is that the model provides a possible explanation for the at-response separation in CPP amplitude (this is also referred to in the Discussion of the main text). We have edited the following sentence in the Supporting Information discussing Fig S11, to touch on your point that CPP build-up rate is also condition-dependent, something which follows logically from the at-response CPP amplitude being condition-dependent but the CPP onset being condition-independent:

"As can be seen, E'(t) shows similar condition-independent late onset and condition-dependent separation in at-response amplitude (and hence also in build-up rate) as we observed for the CPP in our paradigm."

It would help to know more details about the behavior (average RT) to compare to the CPP data.

We are mentioning the range of average response times across conditions, and compare it to the CPP data in the following passage in section "Looming accumulation explains…" in Results:

"… average response times per looming condition in our experiment ranged from 1.1 to 2.4 s, yet it is clear from Fig 3C that the average CPP build-up duration was shorter than 0.4 s for all conditions."

Minor points:

1) Fig. 1, B: the time axis isn't labeled under the response time histograms. Please label

To make it clearer that panels A and B in Figure 1 share the same time axis, we have now placed this time axis between the panels (instead of below panel A, as in the original submission).

2) p. 4: Define \dot{\theta}. It is used before it's defined. Please define first

Thanks. Based on your comment and a related one from Reviewer #3, we have added the following sentence to the first paragraph of the Results section:

"*We denote the projected optical angle of the lead vehicle stimulus on the participant's retina θ, and its optical expansion rate $\dot{\theta} = d\theta/dt$, increasing nonlinearly with time both because of the vehicle acceleration and because the visual angle of an object is (approximately) proportional to the inverse of its distance from the observer.*"

3) p. 5: The statement about "bias" is confusing, I think. Drift is indeed related to evidence accumulation in typical applications of the diffusion model, But "bias" is usually considered a different form of shifting the evidence accumulation process up or down at the start of a trial. It could occasionally be said that the drift is biased, but it sounds odd to me to treat bias and drift as the same thing. I think users of the diffusion model would agree.

This was indeed a misapplication of typical terminology; we have removed mention of the term "bias", since we are not considering this type of initial shift of evidence starting point in our model. Thanks for pointing out our mistake.

4) p. 6: Wow, I did not expect to see evidence in favor of auditory over combined auditory and visual

We are not quite sure what this comment refers to, since there were not really any auditory components of our paradigm.

5) p. 7, Please define "onset" here. How was it computed?

The use of "onset" in that part of the text was in reference to previous papers on the CPP – which is possibly exactly why you asked the question. These previous authors did indeed not study onset directly, so since we are using that term with an exact quantitative meaning in our study, we agree that it may not be appropriate to use it when referring qualitatively to these previous studies. We have removed the use of "onset" in the sentence you refer to, which now reads:

"Second, since in previous studies CPP increase over ERP baseline has been obvious soon after stimulus onset, conditions with slower responses (typically due to less salient stimuli) have produced CPP profiles with build-up commencing earlier in time before the overt response [13–15,17]."

6) I find the word “stringent” to be somewhat unusual. I notice that it sounds unfamiliar to me from the perceptual decision literature. Maybe “powerful” would actually be a better word? The model is powerful because the tests of its predictions are powerful in the statistical sense of power to discriminate between hypotheses.

Yes, we were also not 100% sure stringent was the best word. We would agree that “powerful” is arguably technically a correct term, but to avoid having to argue for its correctness, we have opted instead for "rigorous" in most place, and in the Introduction we instead expanded to say more exactly what we meant: *“detailed models fits of full per-participant probability distributions of response”.*

7) p. 9: "we show that in our paradigm the onset of the CPP, rather than its build-up profile, can be explained by evidence accumulation". It didn't seem to me that the authors investigated the build-up profile of the CPP that closely. Fig. 3C also seems to show something like differences in the CPP buildup profile, to my eye.

As discussed above in (the second part of) our responses to your Major issue 5), it effectively follows from our other analyses that our CPP build-up is condition-dependent, so for this reason we did not analyze our observed build-up rates directly.

We agree, however, that the sentence you quote could be interpreted as saying that we had done a direct analysis, whereas it was rather referring to such analyses in previous work. We have rephrased as follows:

“… in contrast with previous studies on the CPP signature, we show that in our paradigm the late onset of the CPP, rather than a build-up rate present from early on after stimulus presentation, can be explained by evidence accumulation.”

8) p. 10: I like the ending of the Discussion. It seems that this paper calls for a controlled comparison between say, dot motion discrimination, and looming automobile detection. The authors ought to consider doing that comparison in the future. It would really inform the discussion around the CPP and what it reflects.

Thanks for this positive comment. We agree that this would be a very interesting study – it is definitely on our list!

Reviewer #2 comments

This paper describes a nice proof of concept for an early stage evidence accumulation process that depends on optical expansion and later stage decision process that is measured by CPP.

Many thanks for your review. We are glad that you liked the paper.

My one major caution for the conclusions in the paper is that they result from highly flexible models (so goodness of fit is not necessarily an indicator of identifying a useful model of the data) and that the evidence of the distinct stages is still dependent on the assumptions that went into the analysis.

With regards to your first point, about model flexibility and usefulness of the models, we agree that this is an important concern. In the original submission, we briefly touched in the Discussion on the importance of further studying the model's capability of generalizing beyond our dataset, but in the new version we have extended this passage, to now read:

"From an applied perspective, the accumulator models proposed here can be considered as an alternative to the LDT assumption. It should be noted, however, that the focus here was on investigating human ability of collision threat detection in a controlled laboratory experiment, rather than to provide and validate a model for applied use. The alignment with the test track findings by Lamble et al. [52] is encouraging, but further real-world validation, ideally covering a more diversified set of kinematical scenarios, would be advisable."

With regards to your second point, about the possible two-stage accumulation, we agree with you that we are not in a position to draw any strong conclusions at this point, and that we are proposing this idea only as a tentative hypothesis. We tried to make this clear already in the original submission, but we now emphasize it further in the relevant part of the Discussion, which now reads:

"This tentative 'two-accumulator' hypothesis would explain why the onset distributions of the late and rapid CPP in our data can be well accounted for by a looming accumulation model. In the Supplementary Information we provide a computational formulation of this hypothesis and illustrate how it might explain also the at-response CPP separation between looming conditions (Fig 3C, top panel; Fig S11)."

You may also note that the two-stage is not mentioned as one of our four main conclusions in the Discussion, and that we do not mention it at all in the abstract.

I include a few minor comments below.

I would appreciate a bit more clarification on the variables in the study. I suggest making explicit the relationship between response time average optical expansion rate. Also, clarify why that was the independent variable rather than response time.

We used both optical expansion rate and response time as dependent variables (we assume you meant dependent) in our various analyses – but we agree with you that the motivation for this was not clear. We have extended the introductory passage in Results, to now end as follows:

"Note also that in each looming condition there was a direct relationship between response time (the horizontal axis in Figs 1A and B) and optical expansion rate $\dot{\theta}$ at

response (the vertical axis in Figs 1A and C). To align with the existing literature on looming detection, our basic inferential testing on the behavioral data focused on $\dot{\theta}$ at response, whereas to align with the literature on evidence accumulation modeling, our model-fitting was instead focused on the distributions of response time."

Deceleration speed and initial distance were crossed, but there was no indication that their interaction was tested statistically. Why was that test not included?

We did test for this interaction in our main behavioral ANOVA and it was indeed statistically significant, although small in terms of variance explained. Your question made us realize that we had made a mistake in our description of the ANOVA in Materials and Methods, which now correctly states that only first-order interactions were included in the ANOVA model (rather than only second-order interactions, as previously stated – possibly this was one cause for your question). The reason we did not go deeper into reporting the ANOVA results in the initial submission was that our research hypotheses were formulated solely in terms of the main effects of initial distance and deceleration magnitude. For completeness we now provide the full results of this ANOVA in the Supporting information (Table S2).

Figure 1: Although there is a lot already crammed in, there still needs to be axis labels for each subfigure.

We guess that you are referring to panel B, which did not have axis labels in the original submission. To make it clearer that panels A and B in Figure 1 share the same time axis, we have now placed this time axis between the panels (instead of below panel A, as in the original submission).

To what extent is the estimation procedure for the CPP onset novel? There was not a clear justification for this approach in the paper. Was this a planned analysis? How does the uncertainty in the estimates from this procedure influence the downstream analyses (i.e., the accumulator model fits).

These are very valid questions. To address them (and also to address a related comment from Reviewer #1), we have now carried out a sensitivity analysis of our method, reported on in the Supporting Information, and mentioned in the Data acquisition and preprocessing section of Materials and Methods:

"Since our CPP onset estimation method was novel, and not originally planned for, we conducted a sensitivity analysis. As illustrated in Fig S8, this analysis showed that the obtained CPP onset estimates were robust to variations in the parameters of our method.

As described in the Supporting Information, and in our response to the comment from Reviewer #1 above, our sensitivity analysis shows that the main takeaways

from the CPP onset estimation do not change when changing the parameters of our method; the CPP build-up remains late and largely independent of looming condition. This suggests that refitting the accumulator models to the CPP onsets obtained from variations of the estimation procedure should produce similar fits as those we report on in the paper. We have not carried out these refits, however, since doing so would be computationally prohibitive.

It may not be appropriate for this journal, but I suggest adding more detail on the cautionary note about applying the LDT to real-world situations. Is the way LDT used conservative enough that the error does not matter? How would the authors suggest improving the approach?

This is also a good question – but a slightly complex one. The models of collision avoidance response which make use of the LDT assumption, tend to do also assume a fixed (distribution of) response time after the LDT is reached. In our view, also this second assumption is problematic, and we have argued against it elsewhere. In line with what you are saying above, we feel that going deeply into this question is better left for other papers in other journals, but we have added the following entry point into the literature in question in the Discussion:

"Another interesting question for future work is whether these improved models of collision threat detection can support improved models of collision avoidance response. In the road traffic context, some existing models of collision avoidance response suggest that detection and response are separate and sequential steps [57,58,70], whereas other accounts suggest that defensive responses are instead driven directly by kinematical urgency, without a clear role for a first, separate step of detection [58,71,72]."

Pg 10, ln 341: It is not clear how the two stage accumulation process implies that visual looming accumulation occurs without awareness. Please clarify.

Previous work has linked awareness of decision-making with the CPP signature, whereas according to the two-accumulator hypothesis, visual looming accumulation happens before CPP onset. We have tried to make this clearer, modifying the passage in question in the Discussion to read:

"The two-accumulator hypothesis is interesting not least in light of findings that the CPP correlates with subjectively reported experience of the perceptual decision being formed [69]. From this perspective, the late CPP signatures in our data suggest the empirically testable hypothesis that visual looming evidence accumulation (before CPP onset) occurs with near-zero subjective awareness or confidence."

Pg 11, ln 382: How much data was removed?

The full description of the data exclusion is provided in the “Data acquisition and preprocessing” section of Materials and Methods. Based on your comment and a similar one from Reviewer #3, we have added more information on the exclusion also in the main text, in the “Overt responses refute…” section of Results:

“After exclusion of a small minority of trials for early (0.6 %) and missing (0.2 %) detection responses, and a larger number of trials for electrooculographic indications of eye blinks (15.9 %; see Materials and Methods for details), the final data set included 22 participants, with an average of 182 trials per participant (an average of 46 trials per looming condition).”

Do the timeouts vary across levels of the independent variable?

We assume that by timeouts you mean non-responses in looming trials. Since there were only 8 such non-responses throughout the experiment, we did not analyze these further. For your information, there were 4, 0, 2, and 2 non-responses in the different looming conditions, listed in order of decreasing urgency of the looming signal; i.e., there seems to be no clear pattern to these non-responses.

Pg 31, ln 450: Issue with the less than sign.

Thank you – this has been corrected.

Reviewer #3 comments

In this paper, Markkula et al use a combination of computational modeling and human electrophysiology to examine the mechanisms of looming decisions, in a naturalistic task of detecting when a car in front has begun to decelerate. The results show quite convincingly that there is a role for accumulation of optical expansion rate over time in these decisions, where previous research – and policy - has assumed that there is a fixed threshold set on momentary optical expansion rate. They also find that during this task, a centro-parietal ERP signal (CPP) associated with evidence accumulation is strongly present, but, interestingly, has a much more short-lived temporal extent and seems to begin rising a fixed time before the response is made regardless of the strength of the evidence on which the decision is based. The conclusion is thus that evidence accumulation is involved in the looming decision but that in this context, the CPP reflects a second stage of processing after accumulation has reached threshold. I find this an excellent paper, clearly written and with thorough and innovative methods, on a very interesting question that has clear real-world relevance. My comments mostly seek clarifications on how the models were structured and whether the most important alternative accumulation models have been ruled out, none of which harm the central conclusion that these decisions do

involve some form of accumulation over time and not simply a fixed looming threshold.

Many thanks for your review, and for your kind words above.

My first question may simply be out of ignorance of the looming field. But it struck me that what most decision makers might do in this situation – because it seems the more natural thing we do when driving a car – is set a threshold on proximity (a translation of size into an estimate of number of metres away), rather than a rate of optical expansion, because if the car is far enough in front, a very steep rate of optical expansion might not tend to call for braking just yet. I wondered more generally why the "evidence" in this scenario isn't proximity rather than optical expansion rate, and if the decision is re-cast as the former, does that obviate the accumulation since distance is the integral of speed? If my comment is off the mark, it might nevertheless serve to highlight what more general journal audience might think when looking at the situation.

We agree with you that it is not in any way self-evident that a threshold on optical expansion would be a good strategy for deciding when to brake in response to a collision threat on the road. However, quite importantly, the timing of one's *response* a to collision threat is not necessarily strongly related to exactly when one was able to *detect* it, and in our manuscript we are only really dealing with the latter question. Your question is still relevant, of course, and it relates to one of Reviewer #2's comments; as mentioned above we have added the following sentences to the Discussion, to provide an entry point into the bigger literature on this topic:

"Another interesting question for future work is whether these improved models of collision threat detection can support improved models of collision avoidance response. In the road traffic context, some existing models of collision avoidance response suggest that detection and response are separate and sequential steps [52,53,63], whereas other accounts suggest that defensive responses are instead driven directly by kinematical urgency, without a clear role for a first, separate step of detection [53,63,64]."

The most puzzling aspect of the results for me was that the best quantitative fit to the behavioural data did not seem to require either leakage or a threshold set on the evidence. Either of these mechanisms would be able to explain a short-lived evidence accumulation process as suggested by the CPP, and it also seems that either of them is required to fully account for how decisions can be made under such gradual and temporally-uncertain conditions. That is, the winning model has to assume that evidence accumulation begins some fixed time after the onset of the target regardless of the strength of that target, which almost assumes the brain precisely knows the timing of onset in a way that it couldn't possibly, given the timing jitter. A threshold on the evidence would provide a very simple mechanism for

knowing when to kick off the accumulation process, rather like Purcell and Schall's gated accumulator model, and leakage would obviate the need for the accumulator to be kicked off at all – it would just be continuous. For both of these mechanisms, the decision process could be modelled from the beginning of the stimulus and allow naturally for the targets appearing at variable times, and potentially also any effects of that timing (which were not discussed in the paper – were later targets detected differently than earlier ones? See Boubenec et al 2017). But was either model implemented in this way?

Thanks for these thoughts. We thought very similarly about these matters, and were also quite surprised to find that neither sensory thresholds nor evidence leakage were favored in the model comparisons. We did actually model the decision process already from the start of each trial as you suggest above, and to clarify this we have added the following sentence to the Computational models section of Materials and Methods:

"The models were simulated from the start of each trial, i.e., the pre-looming wait time was also simulated, and the accumulator models were initialised at E = 0 for each trial."

With respect to the pre-looming wait time, we had actually already noticed that it had a statistically significant effect on looming detection, but since this effect was so much smaller than the main effects we were targeting in the paper, we did not go further into it in the original submission. Now, in response to your comment here (and comments from the other reviewers), we now provide the full results of the behavioral ANOVA, and we have also added the following sentence to the "Overt responses…" section of Results:

"From a methodological point of view it is worth noting that our behavioral analyses also identified statistically significant effects of experimental block and the 1.5-3.5 s pre-looming wait time. These effects were substantially smaller than the effects of looming condition and between-participant differences (see Table S2), and were therefore not separated out in the subsequent model fitting described below."

To further help make this point clear, we have also made minor additions in "A visual looming…" section in Results and in the sections on model fitting in Materials and Methods.

Furthermore, your comment encouraged us to look more closely at the effect of pre-looming wait time on detection performance, and the model's predictions of the same. We now report on and discuss these results in more detail in the Supporting Information (Fig S3, and also in the "Alternative model variants" section of the SI), and have added the following sentences to the section "A visual looming…" in Results:

"As an additional test of this best-performing model, we also examined its predictions in response to variations in pre-looming wait time. As mentioned above, the model-fitting was blind to this experimental manipulation. However, as shown in Fig S3,

model AV nonetheless predicted the correct pattern of increased looming sensitivity with increased pre-looming wait times, with approximately correct magnitudes."

as well as an additional pointer from the Discussion to the Supporting Information.

Overall, our data thus suggest that an evidence accumulation that is neither gated nor (strongly) leaky is enough to explain the behavior in our paradigm, including the tendency to respond earlier after having waited longer for the looming onset – which the model explains by spurious accumulation toward the decision threshold in the waiting period. This would to us seem to potentially suggest some form of top-down influence on the process implementing the looming evidence accumulation, setting it in a state where it is effectively implementing near-perfect (non-gated and non-damped) drift-diffusion, as for example discussed by Wong and Wang (2006, https://doi.org/10.1523/JNEUROSCI.3733-05.2006, p. 1321 and Supplemental Info section E). This a somewhat complicated discussion, however, which we feel falls outside of the scope of our paper.

I also wondered about the impact of constraining evidence to be positive ("E(i) ≥ 0"), which crops up in the supplemental information (top p4) but not in the main paper and should be more fully explained (ideally, in the mathematical equation, e.g. by using a half-wave rectification operator if that is what is going on).

What we did is not quite half-wave rectification (which implies that the non-rectified signal is available/remembered somewhere in the system), but rather a "reflecting boundary", effectively discarding negative evidence samples if the evidence is already at zero. This was based on the existing literature on evidence accumulation modelling of single-response decisions, where this type of bounding of the accumulator evidence quantity has been common (not least when comparing accumulator evidence to neural signals). We agree that what we did was not sufficiently clear in our original submission, and to improve we have updated Equation (1) in "Computational models" in Materials and Methods with the max function as below:

$$E(i) = \max\left(0, E(i-1) + \tilde{K}\dot{\theta}(i)\,\Delta t + \sigma\nu(i)\sqrt{\Delta t}\right),$$

and added the following sentence:

"Note that we included a reflecting lower boundary at zero (the max function), as often done for evidence accumulators with a single decision boundary [7,20,90,91]."

If evidence samples are not permitted to be negative, then the accumulation of noise alone would build up and eventually cross threshold, because positive values are added to the total but negative ones ignored. Might this contribute to the simulated timecourse of accumulation being longer than it would otherwise be?

As mentioned above, we discard negative evidence samples only if the evidence is already at zero, so the type of buildup of purely positive noise that you are mentioning above does not happen in our model. Purely noise-driven decisions can still of course happen, but that is true for any sufficiently noisy accumulator model.

And there is no provision for the inevitable noisiness of the crossing of a sensory threshold, for instance in a nondecision time variability parameter or by implementing the threshold not on the pure physical signal but the noisy evidence representation itself, with noise sigma and all. My overall point is that given the ability of threshold and leak models to provide a fuller explanation of performance of the task starting from stimulus outset, as well as a short-lived CPP, the details and justification for the specific ways these models were implemented are very important to lay out fully, with perhaps a discussion of alternative ways (e.g. NOT rectifying E(i)).

As mentioned above, we did consider the performance of the task from stimulus outset (i.e., including the pre-looming wait time, as we call it) already in our original submission, and despite this we did not find that threshold(/gated) or leak models could explain the performance better than the model without these assumptions. The new analyses described above, showing that the same model actually also correctly predicted the effect of the pre-looming wait time duration, reinforce this notion even further. We think this addresses the most important part of the point you are making above, and we do hope that with the already mentioned modifications to the manuscript we are now providing sufficient details and justifications for, as well as discussion of, our modeling choices. We agree with you that the further model variants you mention (variability in sensory input or non-decision time, or removing the reflecting evidence boundary) are interesting, but we have not added explicit mention of these possibilities, since the discussion in the main paper and SI is already quite long and sprawling.

Also related to the remarkably short-lived CPP, I wondered what possible impact the fairly strong high pass filter (0.1 Hz cutoff) may have had. This would have the effect of reducing slow shifts in the signals, of the kind that the model predicts but are missing in the real CPPs. It is therefore important to explore the impact of removing the high pass filter.

This is a very good point. We have now rerun the ERP analyses without the 0.1 Hz high pass filter, confirming that the short and late CPP build-up was not due to slower shifts having been filtered away. To also exclude any concerns about the subjective identification of ocular artifact ICA components, we similarly reran the analyses without this step. These results are shown in the Supporting Information (Fig S6). In the main text, we have added the following sentence to the Data acquisition and preprocessing subsection of Materials and Methods:

“The analyses illustrated in Fig 3B and C were also rerun after disabling the 0.1 Hz EEG high-pass filter and the ICA ocular artifact removal, confirming that neither of these two preprocessing steps substantially altered the obtained CPP signatures; see Fig S6.”

More minor:

Drugowitsch et al (2014; eLife) examined decisions about time-varying evidence and might be worth a citation given the authors’ framing in terms of breaking away from stationary evidence

Thanks – we were not previously aware of this paper, which is certainly very relevant here. We are now mentioning it, as well as a couple of more papers that we identified among those citing it, in the Introduction:

*We and others have begun exploring accumulation models of which the input evidence instead scales directly with external sensory data, in tasks such as stick-balancing [31], **visual and vestibular judgment of self-motion [32-34],** longitudinal and lateral control in car driving [29,35–38], and road-crossing decisions [39,40], but these studies have so far not performed model testing and selection at the same level of detail as is typical in the broader evidence accumulation model literature.”*

And in the Discussion:

“Drugowitsch et al. [32,33] provided compelling support for evidence accumulation decision-making in their visual-vestibular heading discrimination paradigm, but did not emphasize detailed fits of response distributions.”

While Drugowitsch et al. did not analyze full probability distributions at the same level of detail that we did, their model analyses were certainly convincing in other respects, prompting us to edit our abstract’s statement on the status of evidence accumulation in sensorimotor tasks from *“not conclusively demonstrated”* to *“with limited in-depth investigation so far”.*

line 83 is written as if it is assumed the reader is already familiar with Lamble et al.

We have edited the sentence in question, to now read:

“Lamble et al. included also non-foveal detection conditions, which we omitted.”

I also suggest finishing the intro or starting the results with a brief intro to the physics of the situation for the uninitiated, e.g. define theta!

We have added the following sentence to the first paragraph of the Results section:

“*We denote the projected optical angle of the lead vehicle stimulus on the participant’s retina θ, and its optical expansion rate $\dot{\theta} = d\theta/dt$, increasing nonlinearly*

with time both because of the vehicle acceleration and because the visual angle of an object is (approximately) proportional to the inverse of its distance from the observer."

I suggest briefly stating in the main part of the results, perhaps in the figure 1 legend, that misses and false alarms were so few as to be not worth showing, because otherwise readers may wonder if the behavioural data are incompletely shown, until they reach the methods, where it is stated in an odd place, in the EEG preprocessing section.

We agree that the mention of misses and false alarms was possibly a bit oddly placed in the Materials and Methods; we have now moved it to the first paragraph in the "Data acquisition and preprocessing" section instead.

In response to your comment, we have also modified the first sentence of "Overt responses refute…", to include mention of the misses and false alarms:

"After exclusion of a small minority of trials for early (0.6 %) and missing (0.2 %) detection responses, and a larger number of trials for electrooculographic indications of eye blinks (15.9 %; see Materials and Methods for details), the final data set included 22 participants, with an average of 182 trials per participant (an average of 46 trials per looming condition)."

This is the same place we learn what proportion of trials were catch trials, which is a detail I believe should be in the results where the task is described as it can be integral to a subject's responding strategy.

We now mention the proportion of catch trials (16.7 %) in the first paragraph of Results.

I may have missed it, but I did not see a statement of the proportion of catch trials that resulted in false alarms, and whether those were included in modelling?

This is a good point, we should have included this information. In the Data acquisition and preprocessing subsection of Materials and Methods we have added the following sentences:

"Out of the 25 x 40 = 1000 catch trials, there were 61 (6.1 %) with false detection responses. The catch trials were not further considered in the analyses or modeling."

In Fig 3C: I suggest adding a note to the legend to explain that the splaying-out of the model simulation's traces across conditions before the response time is a result of post-decision accumulation and that the time of coalescence some ~300 ms before the response marks the point of threshold crossing in the model. Otherwise I

think it may be confusing for some readers that the model has a constant threshold yet the simulated traces reach different levels at response time, until they have sifted through the methods.

We agree, and have added the following sentence to the figure caption:

"Note that the model traces converge at the decision threshold E = 1; the exact location of this point in the plot depends on how much of the non-decision time in the model is assumed to be due to sensory and motor delays, respectively."

Line 227: the "last time ... exceeded?" Is this a typo – should it be the last time 30% was NOT exceeded?

You are of course correct. We have edited this sentence to read:

"We then identified the CPP onset for each averaged trial as the last sample where the averaged response-locked ERP was less than 30 % of its value at the overt response."

The term "pre-decision" is used several times to refer to, e.g. , the response-locked CPP, but I think this will be quite confusing for most, especially when most of the CPP seems to rise after the timepoint the model indicates as the threshold crossing. Perhaps stick to "response-locked," "pre-response" or "pre-commitment"

We agree, and have replaced "pre-decision" with "pre-response", and in some places "pre-decision positivity" with "CPP".
