# Supplementary material for: Accumulation of continuously time-varying sensory evidence constrains neural and behavioral responses in human collision threat detection
Source: PLoS Comput Biol. 2021 Jul 15;17(7):e1009096. doi: 10.1371/journal.pcbi.1009096 (PMC8282001; doi:10.1371/journal.pcbi.1009096)
Supplement: Attachment — Submitted filename: Response to reviewers - Rev2.pdf [file pcbi.1009096.s003.pdf]

The reviewer comments are provided below, together with our responses in blue. The manuscript and the Supporting Information have been provided both in clean versions, as well as in versions where modifications since the previous submission have been highlighted in yellow.

Reviewer 1

I am pretty satisfied with the changes the authors made. First, I want to acknowledge that they were correct in their responses about three mistakes I made in my original review. And then I have one more concern prompted by another reviewer's comments, and one more suggestion about highlighting the optimality properties of CUSUM, and perhaps their own model.

Thank you for your updated review, and for the constructive discussion.

First mistake: I understand now that the evidence/drift is non-constant over time, so there are no closed-form response time distributions.

Second mistake: I'm not sure how I managed to mistake A and AV as standing for Audio and Audio-Visual -- I must've been reviewing another paper with audio and visual conditions at the same time and confused them! Maybe using "VA" instead of "AV" would've prevented that misreading on my part. That's a trivial change that could reduce the chance of other readers making the same silly mistake. It also has the same order as the corresponding words in "variable-gain accumulator".

We considered this possible modification, but since we use the same model denominations throughout the analysis and model code, on balance we felt that the benefit of keeping the same terminology between code and paper outweighed the benefit of avoiding the potential confusion you mention.

Third mistake: Regarding my misinterpretation about whether there were declining thresholds in the evidence accumulator model -- I had to reconstruct why I thought that. It was because of the caption to Fig. 1, parts B and C, in which different final \dot(\theta) values at the time of responding are noted -- these final values decrease for slower looming stimuli. Of course, the time-integral of \dot(\theta) need not be lower when there are lower final \dot(\theta) values. So there's no problem -- I was just confused again. However, since I did make that mistake of interpretation, maybe other readers would too, and it might be worth emphasizing in the caption that the top panel of 1B shows that the integral of the \dot(theta) values in the bottom panel do indeed reach the same fixed threshold at decision time.

Thank you for clarifying. We agree that this could be a source of misinterpretation, and have modified the following sentence in the caption for Fig 1B, to read:

“This model posits that the visual looming evidence shown in panel A is integrated over time together with normally distributed noise, up to a fixed threshold at which detection occurs (example time histories of noisy integration of the looming input shown as thin solid lines, with circle symbols at the fixed decision threshold).

New concern, but not a serious one: I think it's interesting, on my second consideration of this manuscript and of the other reviews, that conceptually, integrating the time-derivative of radial image size over time, without leak, from the start of the trial up to now, is equivalent at every moment to computing the difference between the current radial size and the initial radial size, by the fundamental theorem of calculus. That's only important in the following sense: I can either physically compute a radial velocity, and also compute a running integral of that velocity, or I can take the current size and subtract off my memory of the initial size at the start of the trial. Right? It might seem simpler to some readers to just directly subtract two quantities rather than differentiate, and then undo the differentiation by integrating; and why would the brain use calculus operations when it could just use addition and subtraction?

Personally, I suspect we DO need to compute time derivatives and integrals for all sorts of other reasons, so it's arguably parsimonious for the brain to use differentiation and integration operations. More importantly, the fatal flaw of the pure addition/subtraction approach is that you need to remember an initial radial size, potentially for quite a long time. The velocity computation, in contrast, can be done with a much shorter-duration memory requirement, as in an Euler-method approximation of the derivative -- for that, I just need to remember the radial size from a little while ago in order to subtract it from the current size. And the integration part is really just addition anyway. This might be worth mentioning, if it isn't already mentioned (I couldn't find it).

We agree wholeheartedly with your general point here, as well as your counterargument against its plausibility. It is also known that looming information is potentially estimated as early as the retina (e.g., https://doi.org/10.1126/sciadv.abc9920, and references therein), such that there may not even be a need for the brain to calculate the derivatives you mention. We feel, however, that given the current size of the manuscript and the already sprawling discussion it would be difficult to find space to sensibly discuss these ideas within the present manuscript.

Regarding the reference to Page's CUSUM procedure for change point detection: it would be interesting to analyze more formally to what extent this model implements CUSUM, as has been done for two-boundary drift-diffusion and the sequential probability ratio test. That's not necessary for this paper. However, as I read the description of CUSUM, something, often the likelihood of the data sample (I think?),

is always subtracted off the next data sample before it is added to the running sum. This sounds like a leak term, but I guess it could also just be approximately implemented with a constant inhibition of the accumulator.

Finally, when mentioning CUSUM, I think it would be well worth highlighting the optimality property of CUSUM for certain kinds of scenarios, in terms of minimizing the detection delay of a maximally costly change, while also minimizing the cost of false alarms. Sounds perfect for a case in which the maximally costly change is a deadly collision from failure to detect a vehicle's rapid approach. So there's a reasonable chance that the model here is essentially optimal for maximizing the chance of survival, without also leading to slamming on the brakes every five feet.

We are unsure about your point regarding subtraction of likelihood (we can't find this mentioned in the original 1954 paper at least), but we agree that the potential optimality of this method for looming detection is an interesting topic for future more formal analysis. We have modified the following sentence in the Discussion to read:

"Another interesting question for future work is whether these improved models of collision threat detection (which as mentioned above effectively implement a change detection algorithm which is optimal under certain assumptions [61]) can support improved models of collision avoidance response."

As far as the CPP onsets are concerned, I think the new wording in the paper handles that issue, but I would defer to Reviewer 3 on that.

Please see further below.

Reviewer 2

The revised article reads well and is generally quite clear. The authors have done well addressing my concerns regarding the earlier submission. I apologize for the independent/dependent mix up and now I am going to have to admit making the mistake to my intro psych class.

Many thanks for your second reading of the manuscript, and no apologies needed of course.

Reviewer 3

The authors have provided clear and thoughtful replies to my comments and have made excellent amendments to the manuscript.

On the model specification, it is now clearer how and why it was implemented, and with these clarifications I am satisfied that there aren't obvious alternatives that should have been visited.

Many thanks for your updated review. We are glad to hear that you appreciated the improvements we made in the previous revision.

On the high-pass filter, however, while I do agree that the waveforms are similar to look at when it is removed, I do think it noteworthy that the first half of the timeframe is less flat, and may contain a subtle buildup aspect. A 0.1 Hz low cut off tends to be about as high as you can go without distorting regular, rapidly-fluctuating ERPs, so it stands to reason that it might begin to subtly distort signals as slow as those involved in the current task. I suspect that if you were to apply a similar 0.1 Hz filter on the simulated waveforms from the model, it may appreciably alter the waveforms in a way that makes the buildup more local to the response - not enough to bring it into alignment with the CPP, perhaps, but in principle, it doesn't seem that the comparison can be fully fair when the CPP is subjected to a transformation that attenuates gradual aspects when the simulated waveforms are not.

I therefore suggest replacing the CPP waveforms and onset estimates in the main figure with the unfiltered ones, or at the very least, computing and showing the unfiltered CPP onset estimates similar to main figure 3D, in supplementary Fig S6A.

We do accept your argument here, and have taken a number of steps to address it:

First, in Figure 3 we now show response-locked ERPs both with and without the high-pass filter (panels C and D), so that the reader can see these less pre-processed ERPs directly in the paper itself. The following sentences have been edited accordingly, under "Looming accumulation explains…" in the Results section:

"In contrast, in our response-locked ERP data, there is no obvious separation between conditions in when the CPP build-up commences. Fig 3D shows that the CPP signal is subtly affected by EEG pre-processing choices (most notably, the 0.1 Hz low-pass filter we used causes a slight suppression to negative voltage before the CPP build-up; see further Fig VI in S1 Appendix), but the general aspect of a late, condition-independent CPP onset remains. Meanwhile, as can be seen in Fig 3E, the onset of evidence build-up in our behavioral model was both early and condition-dependent, as expected since the model directly accumulates the looming input."

Second, we applied our existing CPP onset estimation method to the ERPs without high-pass filtering, as you suggested. However, doing so we found that our method effectively relies on the signal having been subjected to prior high-pass filtering; removing this filter introduces an artefactual dependency on looming condition into the CPP onset estimates, because of our approach of defining CPP onset as a fraction of the per-trial at-response ERP amplitude (which varies with looming condition). The high-pass filter mitigates against this problem, because of the slight suppression into negative voltages mentioned above. We now describe this in some detail in the Supplementary Information, with the CPP onset estimates in question shown in Fig VI.D.

Third, we also applied an alternative CPP onset estimation method to the data without high-pass-filtering. This method used an absolute ERP threshold, and we show (Fig VI.E) that this removes the artefactual bias, but introduces other problems.

Essentially, our conclusion here is that our rather simple CPP onset estimation method is far from perfect, and should be improved upon in further work, to ideally allow CPP onset estimation without prior high-pass filtering. Across all these alternative approaches the overall takeaway remains the same, however: Even if we accept as truth the—arguably biased—CPP onset estimates obtained from our original method when applied to the ERPs without high-pass filtering, our CPP signatures still do not align with model evidence build-up in the way that has been shown in previous paradigms. This, in our view, is the key point, rather than the exact onset timings of the CPP.

This takeaway message is further supported by the fourth addition we made to the paper in response to your comment: We refitted our models also to the CPP onsets obtained from the non-high-pass filtered ERPs, and found that, despite the abovementioned added bias, the outcome of the model comparison remained unchanged (model AV outperforms model A, which outperforms model T); see Fig IX.B in the Supplementary Information.

These various additional steps are communicated in the following modified passages in the paper:

At the end of “Looming accumulation explains…” in the Results section:

“The results illustrated in Fig 3F-H were robust to variations in EEG pre-processing and our CPP onset estimation method; see further Materials and Methods, and Figs VI, VIII, and IX in S1 Appendix.”

In the Discussion (added point shown in **bold** here):

“We did not hypothesize in advance that our CPP results would differ from previous findings in this way. For this reason, ***and because the CPP onset analyses we performed here were simple and exploratory in nature****, we are unable to draw any firm conclusions about the underlying reasons for our different-looking CPP signatures.”*

In “Data acquisition and preprocessing” in Materials and Methods (added point shown in **bold** here):

“Since our CPP onset estimation method was novel, and not originally planned for, we also conducted sensitivity analyses. As illustrated in Figs VI and VIII of S1 Appendix, these analyses showed that the obtained CPP onset estimates were robust to variations in the parameters of our method, ***but also that our method was not suitable for use on non high-pass filtered ERP data. Future work can improve on these onset estimation methods, for example along the lines of the approach in [90].”***

Minor:

The abstract says "the model explains CPP onset rather than buildup" but it will not be clear what that means without getting further into the paper - consider whether a tweak here can help to convey that result more clearly, e.g. "The model's estimated decision times align with CPP onset?"

We take your point here, but in our view “align with” suggests something weaker than what we show in the paper. We have tried to make the sentence in question easier to understand by adjusting it to:

“… we show that our model is capable of predicting the onset of the CPP signature rather than its buildup…”
